# Application of 2D polymerase chain reaction for single-tube detection of high-risk human papillomaviruses

**DOI:** 10.3389/fmicb.2025.1528094

**Published:** 2025-01-29

**Authors:** Weifeng Wang, Min Jiang, Ying Liu, Xuan Wu, Yan Chen, Xiaoyun Zhang, Weiwei Liu

**Affiliations:** ^1^Department of Laboratory Medicine, Longhua Hospital, Shanghai University of Traditional Chinese Medicine, Shanghai, China; ^2^Department of Laboratory Medicine, Shanghai Tenth People’s Hospital, Tongji University of Medicine, Shanghai, China; ^3^Department of Pathology, Longhua Hospital, Shanghai University of Traditional Chinese Medicine, Shanghai, China

**Keywords:** human papillomavirus, two-dimensional PCR, cervical cancer, melting temperature, high-throughput

## Abstract

**Purpose:**

The persistent infection of high-risk HPV (HR-HPV) is intricately linked to the onset and progression of cervical cancer. This research endeavored to develop a high-throughput 2D PCR method for closed-tube genotyping of 11 HR-HPVs.

**Methods:**

Base-quenched probes were specifically designed for FAM, VIC, and CY5 channels. The 2D PCR system underwent optimization, with its detection performance assessed in terms of specificity and sensitivity. Plasmid mixtures was used to simulate multiple infections of HPV, providing preliminary insights into the detection efficacy and throughput of the 2D PCR technology. Ultimately, the detection capability of this method was assessed using clinical samples.

**Results:**

The sequenced tags, when paired with primers, could generate Tm differences exceeding 3°C. These were then integrated with a fluorescent channel and Tm to differentiate and identify target genes upon detection. The refined 2D PCR system was confirmed to be free from cross-reactions and exhibited high specificity, capable of detecting 12 target genes within a single tube. A total of 294 cervical exfoliated cell samples were tested using 2D PCR and flow fluorescence hybridization method. The overall concordance between the two detection methods was 96.17% (Kappa = 0.910).

**Conclusion:**

The 2D PCR method, which integrates asymmetric PCR amplification with melting curve analysis, has the capacity to detect 11 types of HR-HPVs across three channels. This closed-tube detection approach offers several benefits including high throughput, straightforward operation, and low detection cost. Consequently, it can be effectively utilized for early screening and prevention of cervical cancer.

## Introduction

Human papillomavirus (HPV) is a pathogen responsible for the most prevalent sexually transmitted infection globally, with a significant lifetime risk of infection among sexually active individuals. Notably, 85% of women and 95% of sexually active men have been infected by HPV (Jain et al., 2023). While the majority of HPV infections are transient, approximately 10% of those who become infected fail to clear the virus through immune responses, resulting in chronic infection that can lead to cancer. In the global female population, cervical cancer ranks as the fourth leading cause of both new cases and deaths, with an estimated 604,000 new cases and 342,000 deaths annually (Sung et al., 2021). China, being a populous developing nation, faces a significant burden of cervical cancer, with approximately 110,000 new cases and 59,000 deaths reported in 2022 (Siegel et al., 2023; Liu et al., 2023). Among the over 200 genotypes of HPV identified to date, only a few are classified as high-risk HPV (HR-HPV) genotypes associated with carcinogenic risks (Kirk and Graham, 2024). In fact, over 90% of cervical cancers can be linked to HR-HPV, with HPV16 and HPV18 contributing to approximately 70% of cervical cancer cases (Skolnik and Morrow, 2023).

HPV infection typically requires several decades to transition normal cells into malignant cells with unrestricted proliferation. This extended timeline offers a significant opportunity for cervical cancer screening (Hu and Ma, 2018). Increasing evidence suggests that molecular detection of HPV is more sensitive and reproducible than cytological screening, offering a five-year long-term assurance for those who test negative (Chen et al., 2024; Dorismond et al., 2024; Heideman et al., 2024). The predominant method of molecular detection involves nucleic acid amplification, primarily through polymerase chain reaction (PCR), followed by open or closed tube detection methods for product identification (Bartosik et al., 2024). Open tube detection methods necessitate the removal of products from the reaction tube and their transfer to other platforms such as sequencing (Nilyanimit et al., 2018), mass spectrometry (Ashley et al., 2018; Fernández Asensio et al., 2018), dot blot hybridization (Kang et al., 2019), among others. These methods demand high-quality instruments and equipment, complex procedures, and most importantly, pose a risk of laboratory contamination due to product exposure. Closed tube detection predominantly relies on TaqMan fluorescent probes for product identification (Troskie et al., 2019; van et al., 2019). Multiplex real-time fluorescence PCR, which utilizes multicolor fluorescent channels, can detect multiple targets in closed tubes. However, in its traditional detection strategy, the number of fluorescent channels, fluorescent probes, and targets to be tested are always equal. This is constrained by the number of fluorescent channels of the PCR machine, limiting the number of targets detectable in a single reaction to no more than six (Hasick et al., 2022). This does not meet the requirements for HR-HPV genotyping. Two-dimensional PCR (2D PCR), which incorporates the melting temperature (Tm) as a second dimension, can augment detection throughput in conjunction with multiplex real-time fluorescence PCR (Li et al., 2021; Huang et al., 2022; Lu et al., 2021). However, employing traditional TaqMan probe technology necessitates the design of specificity probes that match the number of targets. This approach significantly escalates both the fluorescent background and design costs (Huang et al., 2022). The development of an HR-HPV genotyping detection technology, characterized by its simplicity of operation, high detection throughput, and low cost, would inevitably enhance the coverage rate of cervical cancer screening and bolster the efficiency of cervical cancer prevention and treatment.

In addressing these challenges, the field of HPV detection is embracing novel technologies. Zhan et al. introduced a pioneering high-throughput 2D PCR system, leveraging base quenching probe technology, capable of closed tube detection of multiple targets (Zhan et al., 2020). The objective of this study was to employ this 2D PCR technology for the multiplexed typing detection of HR-HPVs, utilizing human hemoglobin subunit beta (HBB) as an internal quality control measure. This approach covered 11 HR-HPVs (HPV16, HPV18, HPV33, HPV39, HPV45, HPV51, HPV52, HPV56, HPV58, HPV59 and HPV68). Upon thorough evaluation, it was determined that the 2D PCR technology was not only user-friendly but also offered a higher detection throughput, stable and reliable detection results. Furthermore, it provided significant economic and social benefits, making it apt for large-scale HPV screening initiatives.

## Materials and methods

### Clinical samples and materials

In this study, we collected a total of 294 cervical exfoliated cell samples from Longhua Hospital affiliated with Shanghai University of Traditional Chinese Medicine and the Tenth People’s Hospital of Shanghai. Of these, 60 samples were determined to be negative for HPV infection, serving as our control group. The remaining 234 samples were found to be infected with 11 high-risk HPV (HR-HPV) types. These infections could be categorized into single, double, or triple infections. The specific primers used in this study were designed by Sangong Biotech Co., Ltd. (Shanghai, China), and the corresponding plasmids were synthesized.

### Design of single pre-tag

This study developed three probe sequences for the prevalent fluorescence detection channels: FAM, VIC, and CY5. The reverse complementary sequences of these three probes served as initial precursor tags. Random positions and numbers of base mutations were introduced to yield three sets of precursor tag sequences that retained homology with the original tags but varied among themselves. Their homology was further confirmed or ruled out using BLAST. These precursor tag sequences ranged in length from 20 to 30 base pairs. Given that the G base in single-stranded DNA exhibits the highest quenching efficiency (Mao et al., 2018), additional G bases were incorporated at the 5′ end of the precursor tag sequence to amplify the quenching effect on the fluorescent group and enhance the stability of the precursor tag. To ensure suitable Tm differences among the precursor tag sequences, we combined the base quenching probes with synthesized precursor tags and conducted a melting curve analysis. The precursor tags were found to bind directly with the base quenching probes. Based on the Tm span of each set of precursor tags, the Tm difference between adjacent sequences, and the quality of the melting curves, we ultimately selected 12 precursor tag sequences from the three sets for the construction of a 2D PCR tag library (Supplementary Table 1).

### Design of HPV genotypes primer

The 2D PCR method employs an asymmetric amplification format, where only one of the target-specific primers incorporates a tag sequence (hereafter referred to as the “tagged primer”), which serves as the amplification template. The other primer, devoid of the tag sequence (hereafter referred to as the “untagged primer”), amplifies the pre-tag sequence under its influence. Initially, we conducted a comprehensive search of sequences for 11 types of HR-HPV (16, 18, 33, 39, 45, 51, 52, 56, 58, 59, 68) and Hemoglobin subunit beta (HBB) genes as an reference gene in the NCBI database. By integrating the results of our literature research with sequence comparisons using Clustal software, we were able to identify the conserved sequences of each target gene. We then utilized the Primer-BLAST function of the NCBI database to design 12 pairs of target-specific primers. The Tm value, GC content, hairpin structure, and dimer structure of these designed primers were evaluated using OLIGO 6 software to ensure that the primer design met the experimental requirements. Following the exclusion of non-specific reactions between primers, targets, and probe sequences via the NCBI database BLAST function, the reverse complementary sequences (tag sequences) of F1, F2, F6, F10, and F15 were, respectively, attached to the 5′ end of the upstream primers for HPV18, HPV58, HBB, HPV33, and HPV16. Similarly, the reverse complementary sequences (tag sequences) of V1, V2, V7, and V8 were linked to the 5′ end of the upstream primers for HPV52, HPV45, HPV51, and HPV39. Finally, the reverse complementary sequences (tag sequences) of C4, C7, and C9 were connected to the 5′ end of the upstream primers for HPV59, HPV68, and HPV56. Primers specifically designed for this experiment primarily targeted the E1, E4, and L1 regions of the HPV genome. The sequences and amplification sites of these primers are detailed in Supplementary Table 2.

### Sensitivity analysis

The plasmid stock solution, with a concentration of 10^12^ copies/μL, was initially diluted in a tenfold gradient to reach a concentration of 10^1^ copies/μL. For the purpose of LOD validation, plasmids were selected that ranged in concentrations from 10^1^ copies/μL to 10^8^ copies/μL. It is important to note that the LOD for each target gene must be ascertained independently. The 2D PCR system operates by sequentially detecting single plasmids at varying concentrations. The lowest concentration at which the target gene can be identified is defined as the LOD for that specific target gene.

### Design of 2D PCR reaction system

The 2D PCR system encompasses three types of base quenching probes, twelve pairs of tagged primers, and untagged primers. To augment the reaction’s specificity, the system utilizes heat-start HS Taq DNA polymerase. Prior to implementing multiplex detection, this study initially employed the 2D PCR system to examine a single target gene, thereby preliminarily assessing the feasibility of the 2D PCR system’s design. Following verification and system optimization, the final total reaction system is detailed in Supplementary Table 3.

### Statistical analysis

When analyzing clinical samples, the outcomes of 2D PCR and flow fluorescence hybridization methods should be interpreted separately. Any samples that tested negative with internal reference genes were excluded from the study. Given the varying range of HPV genotypes covered by these two techniques, we only documented the detection results for the 11 shared HR-HPVs due to the necessity for statistical analysis. The concordance between flow fluorescence hybridization and 2D PCR was evaluated using Cohen’s Kappa coefficient, calculated via SPSS version 24.0 (IBM Corporation, USA).

## Results

### Validation results of single pre-tag Tm

**Figure 1:**
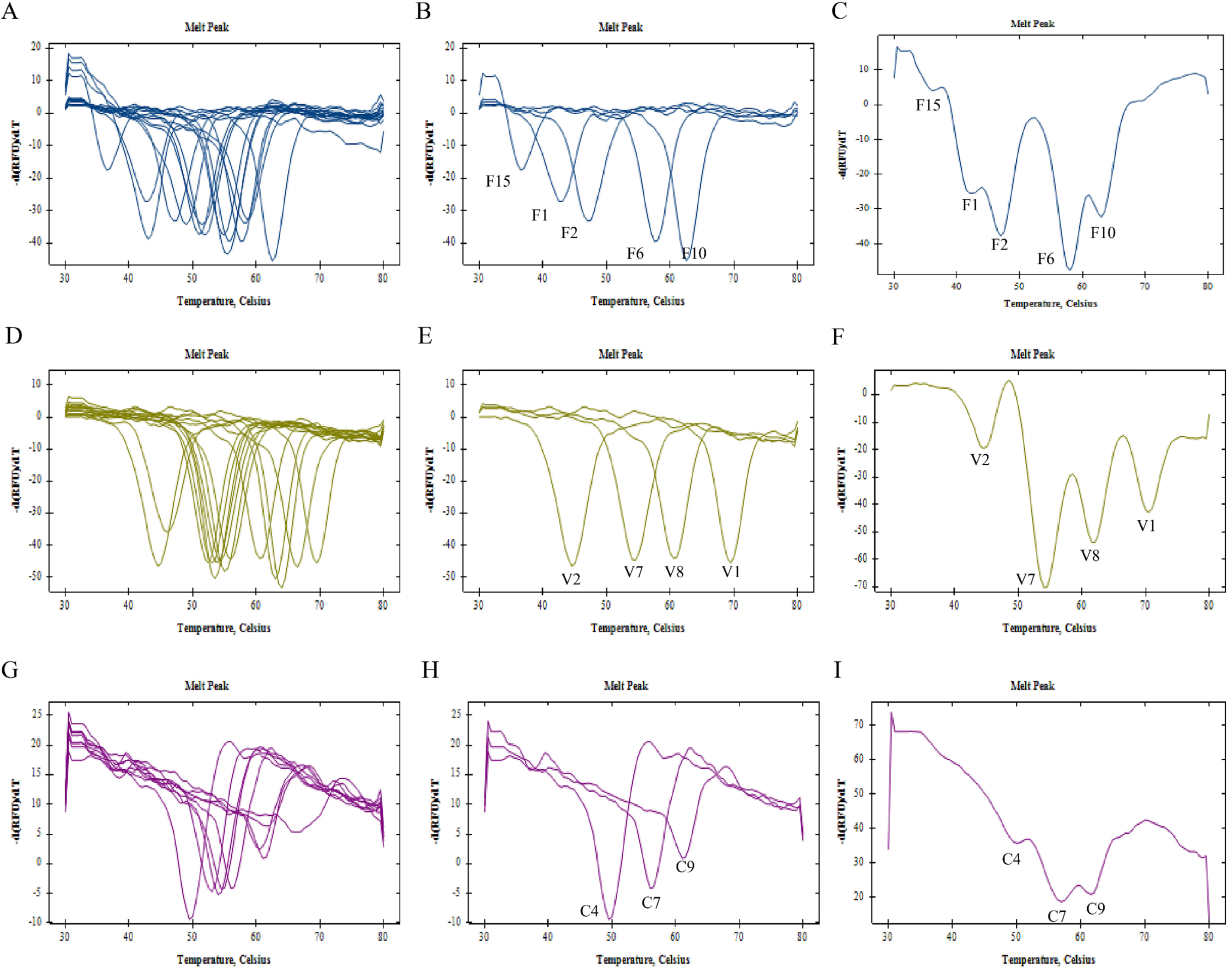
Establishment of 2D PCR tag library. **(A)** Melting curve summary of all the pre-tags of FAM channel and **(B)** selected pre-tags F1, F2, F6, F10, F15 of FAM channel, **(C)** melting curve analysis of mixed run of selected pre-tags F1, F2, F6, F10, F15 of FAM channel; **(D)** melting curve summary of all the pre-tags of VIC channel and **(E)** selected pre-tags V1, V2, V7, V8 of VIC channel; **(F)** melting curve analysis of mixed run of selected pre-tags V1, V2, V7, V8 of VIC channel; **(G)** melting curve summary of all the pre-tags of CY5 channel and **(H)** selected pre-tags C4, C7, C9 of CY5 channel; **(I)** melting curve analysis of mixed run of selected pre-tags C4, C7, C9 of CY5 channel.

In this study, we designed a total of 15 FAM channel pre-tag sequences, 14 VIC channel pre-tag sequences, and 9 CY5 channel pre-tag sequences. Upon verification, all synthesized pre-tags demonstrated complementary binding with the corresponding fluorescence channel’s base quenching probe under low temperature conditions. Furthermore, these pre-tags dissociated from the probe during the heating process, resulting in a specific single melting peak (Figures 1A,D,G).

As illustrated in Table 1, the Tm range for pre-tag groups within the FAM channel was established between 36.5–62.5°C. For the VIC channel, this range extended from 44.5–69.5°C, and for the CY5 channel, it spanned from 49.5–66.0°C. To ensure clear differentiation between adjacent melting peaks, a screening criterion was applied: pre-tag groups within each channel should have a minimum Tm difference of 3°C. By integrating the Tm values of these pre-tag groups with the quality of individual pre-tag melting curves (F1 at 42°C, F2 at 47°C, F6 at 57°C, F10 at 62°C, and F15 at 37°C), we initially selected these values to construct the label library for the FAM channel. Similarly, V1 at 69°C, V2 at 44°C, V7 at 55°C, and V8 at 60°C were chosen to establish the label library for the VIC channel. Finally, C4 at 50°C, C7 at 56°C, and C9 at 62°C were selected to create the label library for the CY5 channel (Figures 1B,E,H).

**Table 1:** Comparison of consistency between flow fluorescence hybridization and 2D PCR.

	Flow fluorescence Hybridization assay Positive samples		Flow fluorescence Hybridization assay Negative samples			
HPV types	2D PCR Positive samples	2D PCR Negative samples	2D PCR Positive samples	2D PCR Negative samples	Coincidence rate(%)	Kappa
HR-HPV	226	8	1	59	96.17	0.910
HPV16	28	6	0	260	82.35	0.892
HPV58	26	3	0	265	89.66	0.940
HPV18	27	0	0	267	100.00	1.000
HPV52	43	1	0	250	97.73	0.987
HPV51	27	0	0	267	100.00	1.000
Other HPV^a^	95	8	11	180	83.33	0.859

^aHPV33, HPV39, HPV45, HPV56, HPV59 and HPV68 with low infection frequency were analyzed.^

In order to preliminarily investigate the viability of multiplex detection, we amalgamated the pre-tag groups utilized for library construction in a single channel at an equal ratio. The analysis of the melting curve results indicated that the number of melting peaks generated under the mixed state was equivalent to the quantity of pre-tags present in the system. This number could correspond to a unique one, as per Tm (Figures 1C,F,I). This finding substantiated that the initial selection of three sets of pre-tag sequences could be effectively utilized to construct a two-dimensional PCR label library.

### Optimization of F/R primers, Mg^2+^ and probe conditions

The PCR conditions were meticulously optimized for the tagged primers. Subsequently, 0.05 μL, 0.1 μL, 0.15 μL, and 0.2 μL of each target gene-tagged primer were introduced to observe variations in the melting curve upon detection of a single plasmid. The change in the melting curve when detecting a single plasmid should then be observed. The quantity of tagged primer incorporated is consistently maintained at 0.1 μL, while the volume of untagged primer is sequentially adjusted to 0.2 μL, 0.4 μL, 0.6 μL, and 0.8 μL, in order to ascertain the optimal quantity of untagged primer required. Subsequently, based on the optimal quantity of amplicons, we incorporated a base quenching probe, tagged primer, and untagged primer into the 2D PCR reaction system. We adjusted the Mg^2+^ concentration in the system to 1 mM, 1.5 mM, 2 mM, and 2.5 mM respectively, and then conducted detection for single plasmid analysis. Finally, based on the optimization results, adjustments were made to the quantities of untagged and tagged primers. Additionally, the quantity of three base quenching probes was incrementally increased during the detection of a single plasmid. This was done by observing the dynamic changes in the melting curve corresponding to probe loading amounts of 0.1 μL, 0.2 μL, 0.3 μL, 0.4 μL, and 0.5 μL, respectively, (Supplementary Figure 1).

### Specificity verification experiment result

**Figure 2:**
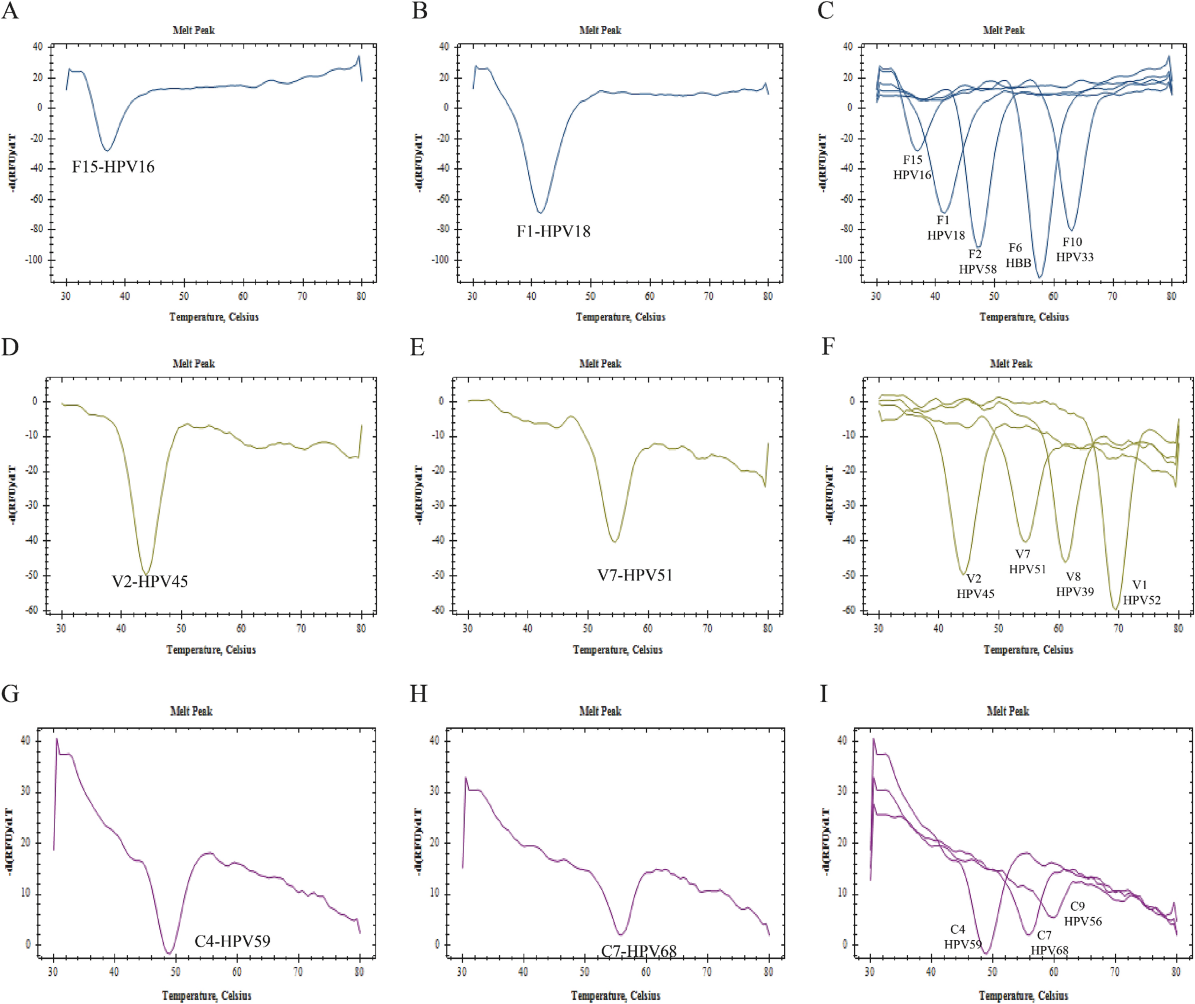
Specificity verification. All figures displayed only a single melting curve, with no evidence of cross-reaction.

Figure 2 presents the specificity verification results. It is evident from this figure that there was no cross-reaction between primers, probes, and gene sequences within the 2D PCR system. In each single gene detection, only one primer was capable of amplifying the target sequence, and a singular probe could recognize the amplified product. Furthermore, all melting curves exhibited a single peak. A summary of these findings can be seen in Figure 2. The significant difference in Tm value across each detection channel was easily discernible. When combined with fluorescence channel and Tm values, all target genes were successfully identified: HPV16 (FAM-37°C), HPV18 (FAM-42°C), HPV33 (FAM-62°C), HPV39 (VIC-60°C), HPV45 (VIC-44°C), HPV51 (VIC-55°C), HPV52 (VIC-69°C), HPV56 (CY5-62°C), HPV58 (FAM-47°C), HPV59 (CY5-50°C), HPV68 (CY5-56°C), HBB (FAM-57°C). Notably, when samples tested positive for HPV6, HPV61, or HPV81 via flow fluorescent hybridization assay, only a melting peak representing the HBB gene was observed in the 2D PCR melting curve. This suggests that there was no cross-reaction between the 2D PCR and low risk subtypes of HPV6, HPV61, and HPV81. Overall, these experimental results demonstrate superior specificity of the 2D PCR method.

### LOD verification experiment result

**Figure 3:**
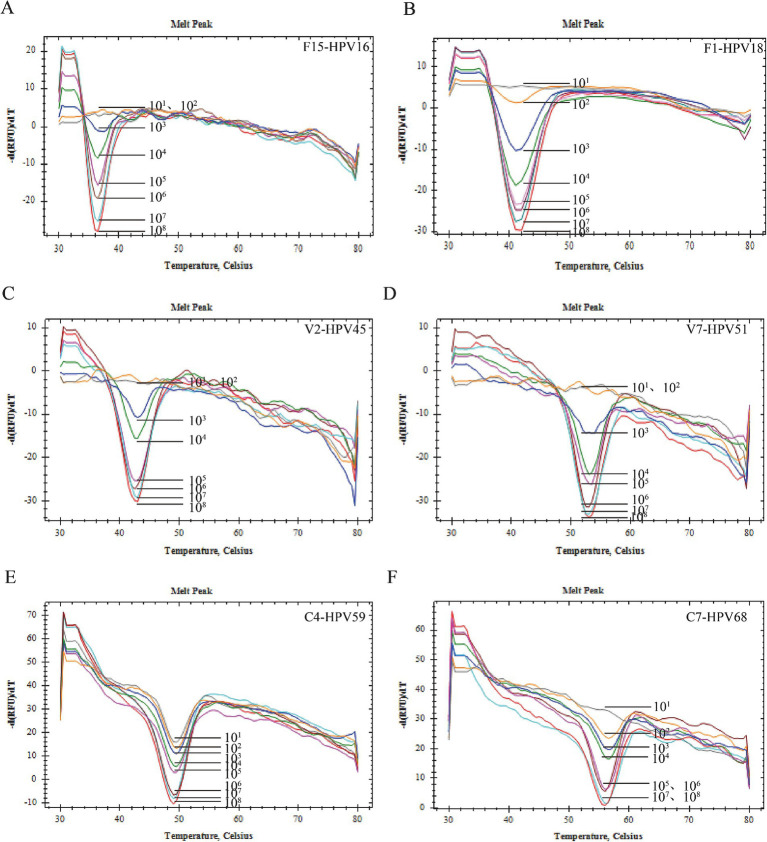
Sensibility verification. **(A)** The sensitivity of HPV-16 was 10^3^ copies/reaction. **(B)** The sensitivity of HPV-18 was 10^2^ copies/reaction. **(C)** The sensitivity of HPV-45 was 10^3^ copies/reaction. **(D)** The sensitivity of HPV-51 was 10^3^ copies/reaction. **(E)** The sensitivity of HPV-59 was 10^1^ copies/reaction. **(F)** The sensitivity of HPV-68 was 10^2^ copies/reaction.

Plasmids with concentrations ranging from 10^1^ copies/μL to 10^8^ copies/μL were utilized to ascertain the Limit of Detection (LOD). Within the detectable concentration range, it was observed that the peak of the melting peak escalated in correlation with an increase in plasmid concentration. The LOD for HPV39 and HPV59 was established at 10^1^ copies/μL (0.01 pg./mL), while for HPV18, HBB, and HPV68, it was determined to be 10^2^ copies/μL (0.1 pg./mL). For HPV16, HPV33, HPV58, HPV45, and HPV51, the LOD was found to be 10^3^ copies/μL (1 pg./mL). Furthermore, the LOD for HPV52 was established at 10^4^ copies/μL (10 pg./mL), while for HPV56, it was determined to be 10^5^ copies/μL, equivalent to 100 pg./mL (Figure 3).

### Multiple infection simulation experiment

**Figure 4:**
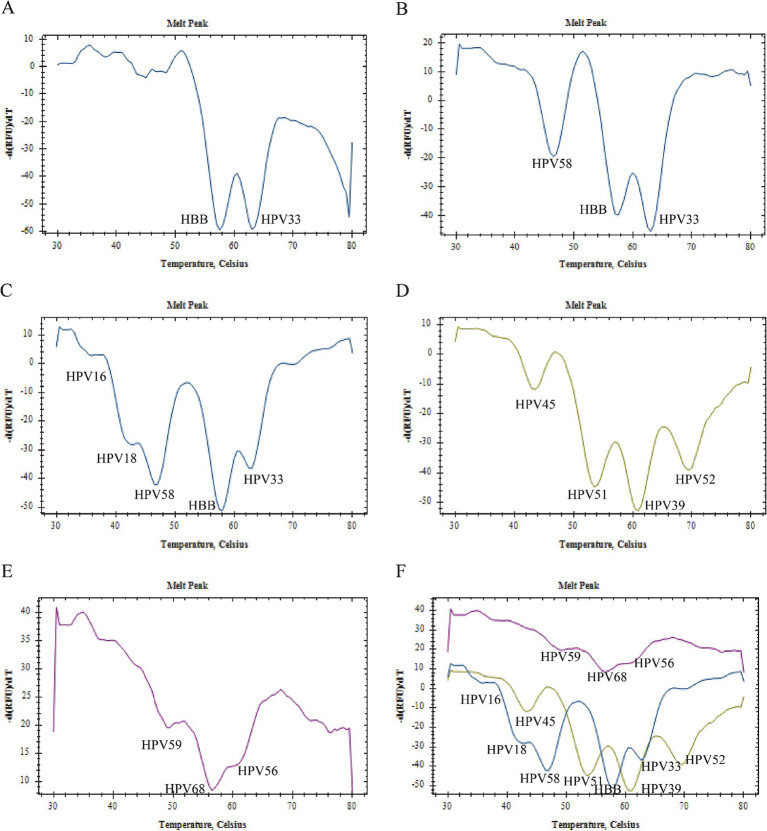
Multiple infection simulation. **(A)** Melting curve of FAM channel in HPV33 single infection; **(B)** Melting curve of FAM channel in HPV33, HPV58 double infection; **(C–F)** are the summary maps of melting curves of FAM channel, VIC channel, CY5 channel, respectively, for detection of 12 target genes by 2D PCR in a single tube.

As depicted in Figure 4, the FAM channel melting curve from a single infection simulation involving both the HPV33 and HBB plasmids exhibited dual peaks, which were subsequently identified as HPV33 and HBB based on their Tm values. In contrast, the FAM channel melting curve from a dual infection simulation incorporating the HPV33, HPV58, and HBB plasmids revealed three distinct melting peaks, corresponding to HPV33, HPV58, and HBB based on their Tm values. Upon analyzing hybrid DNA from 12 different plasmids, the melting curves of three detection channels yielded a total of 12 melting peaks, each corresponding to one of the 12 target genes amplified by 2D PCR. These findings suggest that 2D PCR possesses the capability to simultaneously detect 2, 3, and even 12 target genes, identifying up to 11 HR-HPVs in a single run.

### Clinical sample testing

A total of 294 cervical exfoliated cell samples were collected in this study, and the extracted DNA was detected by flow cytometry and 2D PCR.

Flow cytometric fluorescence hybridization revealed no instances of HPV infection in the 60 samples. However, 234 samples were found to be infected with 11 types of HR-HPV, as identified by 2D PCR. These infections could be categorized into single infections (198, or 84.62%), double infections (34, or 14.53%), and triple infections (2, or 0.85%). Among these, 34 samples tested positive for HPV16, 27 for HPV18, 11 for HPV33, 22 for HPV39, 18 for HPV45, 27 for HPV51, 44 for HPV52, 23 for HPV56, 29 for HPV58, 23 for HPV59, and 14 for HPV68.

**Figure 5:**
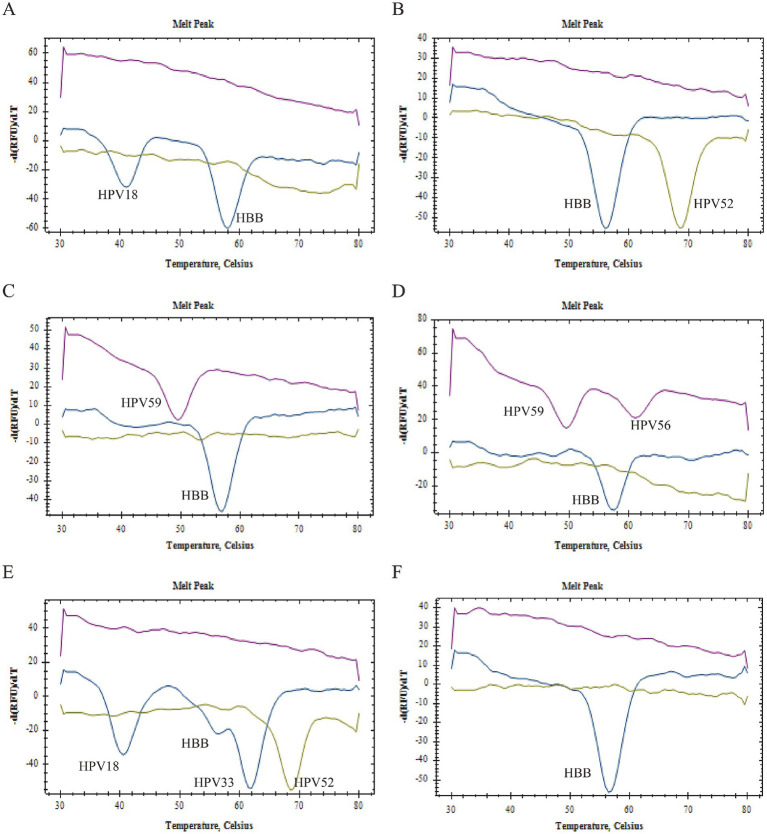
Clinical sample testing. **(A)** Samples were detected as HPV16 single infection. **(B)** Samples were detected as HPV52 single infection. **(C)** Samples were detected as HPV59 single infection. **(D)** Samples were detected as HPV52, HPV58 double infection; **(E)** Samples were detected as HPV16, HPV39, HPV68 triple infection. **(F)** Samples were detected as HPV negative.

The 2D PCR identified 227 positive samples and 67 negative samples. The positive samples were further categorized into 193 (85.02%) single infections, 30 (13.22%) double infections, and 4(1.76%) triple infections. These included 28 HPV16 positives, 27 HPV18 positives, 16 HPV33 positives, 25 HPV39 positives, 18 HPV45 positives, 27 HPV51 positives, 43 HPV52 positives, 20 HPV56 positives, 26 HPV58 positives, 21 HPV59 positives, and 14 HPV68 positives. Figure 5 illustrates the detection results of various clinical samples using the 2D PCR, including both negative samples and samples with single or multiple HPV infections.

### Consistency analysis

Table 1 conducted a consistency analysis of the sample detection results. Among the 294 clinical samples that were co-detected, there were eight samples reported as positive by flow fluorescence hybridization and negative by 2D PCR. Additionally, one sample was reported as positive by 2D PCR and negative by flow fluorescence hybridization. The overall detection result agreement rate between these two methods was 96.17% (Kappa = 0.910). In terms of HPV typing results, the consistency between flow fluorescence hybridization and 2D PCR for detecting HPV18 and HPV51 was remarkably high, with an agreement rate of 100.00% (Kappa = 1.000). However, this rate decreased to 82.35% (Kappa = 0.892) for HPV16.

## Discussion

This study developed a high-throughput 2D PCR system utilizing base quenching probe technology, identifying 12 target genes in FAM, CY5, and VIC channels. The research synthesized three non-homologous probe sequences and identified three groups of pre-tagged sequences with a temperature difference (Tm) exceeding 3°C. The reverse complementary sequence of these pre-tagged sequences was then ligated to the upstream primer’s 5′ end, specifically directing toward the target gene under examination. Following system optimization experiments, we ultimately designed a 2D PCR system comprising three types of base quenching probes, 12 band tagged primers, and 12 untagged primers. Verification confirmed that the optimized system exhibited excellent specificity and sensitivity, capable of simultaneously detecting all 12 target genes in a single reaction. This system holds significant potential for detecting multiple infections of HPV and can provide reliable detection results for clinical samples.

In comparison to existing HPV detection methods, 2D PCR technology presents a high-throughput closed tube, whole typing detection approach. This method can identify 11 HR-HPV types associated with cervical cancer solely through PCR amplification and melting curve analysis, eliminating the need for additional product identification equipment or complex processing procedures. It also mitigates the risk of false positives due to laboratory contamination. While it encompasses fewer HPV genotypes than flow fluorescence hybridization, 2D PCR offers superior economic benefits. Its ability to detect multiple genes in a single channel necessitates only one fluorescent probe, which does not require quenching groups. According to Luo et al., the estimated total cost of Real-time quantitative PCR (Q-PCR) for a single reaction is approximately $0.055, encompassing the costs of primers, probes, buffer, dNTPs, and Taq DNA polymerase (Luo et al., 2009). In the 2D PCR method, 5 OD of primer can detect approximately 2000 DNA samples. When calculating based on 30 pairs of HPV primers, the cost is roughly $1.42 per sample. In contrast, the cost of the flow fluorescence hybridization assay (Shanghai Topview Life Technology Co., LTD) is approximately $15 per sample. The cost of detection is approximately one-tenth that of flow fluorescence hybridization. Moreover, the 2D PCR analysis technology offers simplicity in operation, requires basic equipment, and does not necessitate additional product identification equipment or complex processing procedures, making it easily accessible and adaptable. Furthermore, the system design of 2D PCR is more straightforward and adaptable. Both probes and labels do not directly target the gene under examination; instead, they are randomly designed. This allows for an extensive range of available probe and label sequences, reducing the complexity of design due to sequence similarity with the target gene. The 2D PCR technology has demonstrated exceptional performance in high-throughput detection. When identifying an equal number of target genes, its reaction time is shorter, results are reliable and stable, and the probe recognition function remains unaffected by genomic mutations in the amplified region. This makes it suitable for large batch population screening work. Therefore, 2D PCR is an extremely viable and promising HPV detection technology, aiding in early detection of HPV and prevention of cervical cancer screening.

We do Sanger sequencing for all the same samples as we analysed with 2D-PCR. Our analysis revealed that, of the 294 samples in our dataset, the sensitivity and specificity of the 2D-PCR method compared to Sanger sequencing were 96.6 and 90.8%, respectively. In contrast, the sensitivity and specificity of the flow fluorescence hybridization assay were 99.6 and 79.7%, respectively. It is well-established that the sensitivity of 2D-PCR closely mirrors that of flow fluorescence hybridization, while offering superior specificity. The flow fluorescence hybridization method yielded inconsistent results with sequencing in 16 samples, while the 2D PCR produced discrepancies in 14 samples. The concordance rate between the flow fluorescence hybridization method and sequencing stood at 93.9%, whereas the concordance rate between 2D PCR and sequencing was slightly higher, at 94.7%. These findings suggest that the 2D PCR method offers a performance advantage over existing commercial HPV tests.

Among the existing literature on 2D PCR technology, the HPV detection system developed in this study incorporated the highest number of target genes for testing. It was also the first to demonstrate the potential of 2D PCR technology for simultaneous detection of 12 target genes. However, there is still room for enhancement in its detection performance. In the LOD verification experiments, low concentrations of HPV52 and HPV56 plasmids were not readily detected. When clinical samples were analyzed, the melting peak of HPV16 tended to be flat, failing to meet the interpretation criteria for positive results. This discrepancy between 2D PCR and flow fluorescence hybridization methods, as well as sequencing, can be attributed to this issue. The single pre-tag analysis, mixed pre-tag analysis, and single pair primer detection experiments conducted in this study yielded melting peaks with varying relative peaks. These findings suggest that the quality of the melting curve and the detection of target genes are influenced by the amplification efficiency of primers and the binding ability of pre-tags to probes. If a higher amplification efficiency primer and a more strongly binding pre-tag can be designed for the 2D PCR system, changes in fluorescent signal will be more easily detected. This would facilitate the detection of low concentration DNA and improve the quality of the melting curve. Therefore, it is anticipated that the performance indicators and application value of 2D PCR will continue to be optimized.

The essential elements of the 2D PCR system comprise the base quenching probe and homologous tag sequences. A single tag lacks target gene specificity; however, when coupled with the target gene primer, the tag can specifically refer to the target gene under examination based on the Tm value of the reverse complementary sequence. Thus, provided there is no non-specific binding among the probe sequence, tag sequence, target sequence, and primer sequence, the base quenching probe and tag sequence developed in this study can be effectively used for high-throughput detection of other pathogen target genes, indicating wide applicability and flexibility.

Furthermore, this research employed a point mutation strategy to modify the Tm value of the pre-tag sequence. The effective hybridization length between the pre-tag and probe can also induce a shift in the Tm value during the melting curve analysis process. This approach not only streamlines the design of fluorescent probes and homologous labels but also yields Tm values that are closer to their true values (Xu et al., 2023). 2D PCR technology offers substantial social and economic advantages, demonstrating strong clinical application potential and transformative capabilities. If further refinements and optimizations are achieved, it is anticipated that this technology will assume a more pivotal role in high-throughput detection.

## Data Availability

The original contributions presented in the study are included in the article/Supplementary material, further inquiries can be directed to the corresponding authors.
