# Supplementary material for: Application of 2D polymerase chain reaction for single-tube detection of high-risk human papillomaviruses
Source: Front Microbiol. 2025 Jan 29;16:1528094. doi: 10.3389/fmicb.2025.1528094 (PMC11815664; doi:10.3389/fmicb.2025.1528094)
Supplement: Supplementary file 1 [file Data_Sheet_1.doc]

Supplementary Figure 1 Condition optimization


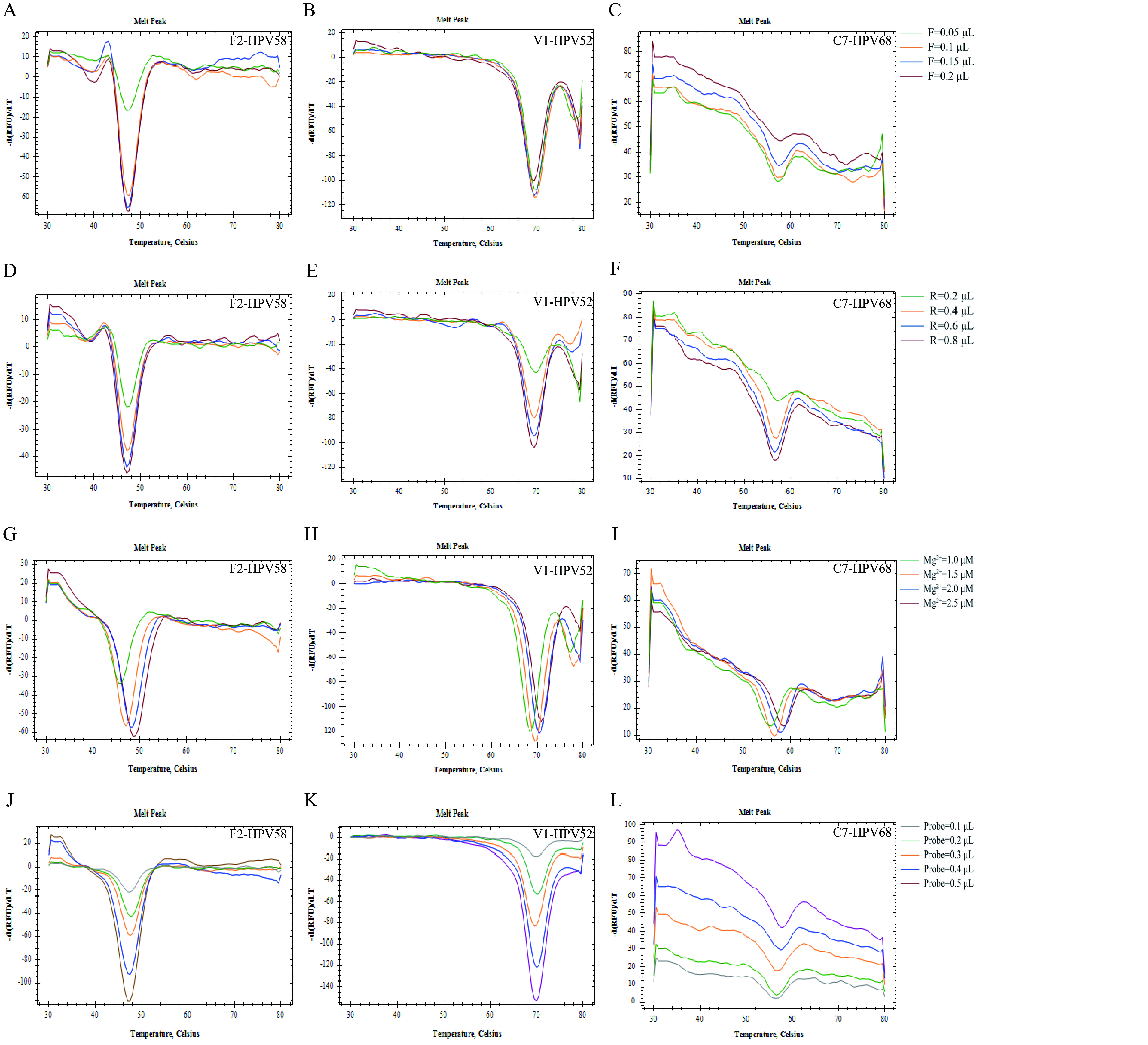


(A) Melting profiles of FAM channel HPV58, (B) VIC channel HPV52, (C) CY5 channel HPV68 with tagged primers at amplicon addition amounts of 0.05 μL (green), 0.1 μL (orange), 0.15 μL (blue), and 0.2 μL (red), respectively, and HPV58, HPV52, HPV68 without tagged primers at an amplicon addition amount of 0.6 μL. (D) Melting profiles of FAM channel HPV58, (E) VIC channel HPV52, (F) CY5 channel HPV68 without tagged primers at an amplicon addition amount of 0.2 μL (green), 0.4 μL (orange), 0.6 μL (blue), and 0.8 μL (red), respectively, and HPV58, HPV52, HPV68 with tagged primers at an amplicon addition amount of 0.1 μL. (G) Melting profiles of FAM channel HPV58, (H) VIC channel HPV52, (I) CY5 channel HPV68 with Mg^2+^ concentrations of 1 mM (green), 1.5 mM (orange), 2 mM (blue), and 2.5 mM (red). (J) Melting profiles of FAM channel HPV58, (K) VIC channel HPV52, (L) CY5 channel HPV68 with fluorescent probes at an amplicon addition amount of 0.1 μL (gray), 0.2 μL (green), 0.3 μL (orange), 0.4 μL (blue), and 0.5 μL (red).

Supplementary Table1 Summary of Tm of pre-tag sequences

| Name | Pre-tag(5’-3’) | Tm(℃) |
| --- | --- | --- |
| F1 | GGGGG GTGGAAATGTATAAGCTAGGTAATGG | 42 |
| F2 | GGGGG GTGGAAGTGCTTAAGGTTCGTAATGG | 47 |
| F6 | GGGGG GTGGAAGTGTATAAGGTAGGTAATGG | 57 |
| F10 | GGGGG GTGGAAGTGTATAAGGTTGGTAATGG | 62 |
| F15 | GGGGG GTGGCAGCGCATGAGGTTGGTCATGG | 37 |
| V1 | GGGGG GTGAAAGGGGTGGAGGTTGAGAGTTAGGG | 69 |
| V2 | GGGGG GTGAAAGGAATGGAGATTGAAAGTTAGGG | 44 |
| V7 | GGGGG GTGAAACGGGTTGAGGTTGGGAGTTAGGG | 55 |
| V8 | GGGGG GTGAAAGGGGTGCTGGTTGAGAGTTAGGG | 60 |
| C4 | GGGGG GTAGAGAGTCCCAAGGGGAACAGACTAAGAG | 50 |
| C7 | GGGGG GTAGAGAGTACCAACGGGAAGTGACTAAGAG | 56 |
| C9 | GGGGG GTAGAGAGAACCAAGGGCAAGTGACTAAGAG | 62 |

Supplementary Table2 Probe primer sequence

| Genotype | Mismatched targets | Primer sequence (5′–3′) | Genome position | Length of the amplimer | Tm(℃) |
| --- | --- | --- | --- | --- | --- |
| FAM | CCATTACCAACCTTATACACTTCCAC | Universal probe |  |  |  |
| HPV-18 | CCATTACCTAGCTTATACATTTCCAC | F:GACAGCACAGGCATTGTTCCAT | E1：192-213 | 121 bp | 42 |
|  |  | R:CTCCAGCCGCTCCCCTAAT | E1：312-294 |  |  |
| HPV-58 | CCATTACGAACCTTAAGCACTTCCAC | F:ATGATAGATGATGTAACAGCCATAAGC | E1：1537-1563 | 109 bp | 47 |
|  |  | R:GTACTAATGCCCTATGTTTTACATCTATTG | E1：1645-1616 |  |  |
| HBB | CCATTACCTACCTTATACACTTCCAC | F:AGGTTCTTTGAGTCCTTTGGGG | HBB：251-272 | 126 bp | 57 |
|  |  | R:GAGGTTGTCCAGGTGAGCCAG | HBB：376-356 |  |  |
| HPV-33 | CCATTACCAACCTTATACACTTCCAC | F:GGCATGTGTAGGCCTTGAA | L1：303-321 | 101 bp | 62 |
|  |  | R:TGTTACTGGTTTCAGTGTCATCAA | L1：403-380 |  |  |
| HPV-16 | CCATGACCAACCTCATGCGCTGCCAC | F:TAATTCACAGGCAAAAATTGTAAAGG | E1：1185-1210 | 98 bp | 37 |
|  |  | R:ATTTTATCCATTGACTCATACTCATTTGT | E1：1282-1254 |  |  |
| VIC | CCCTAACTCTCAACCTCCACCCCTTTCAC | Universal probe |  |  |  |
| HPV-52 | CCCTAACTCTCAACCTCCACCCCTTTCAC | F:CATTTCAGAGGACGAGGATG | E4：93-112 | 101 bp | 69 |
|  |  | R:CCTCATGTTCTGCCTGTTCA | E4：193-174 |  |  |
| HPV-45 | CCCTAACTTTCAATCTCCATTCCTTTCAC | F:ACTGCCAAGCCAAATATTTAAAAGA | E1：1166-1190 | 132 bp | 44 |
|  |  | R:TCCAATCCCCACCTTCATCTATTTT | E1：1297-1273 |  |  |
| HPV-51 | CCCTAACTCCCAACCTCAACCCGTTTCAC | F:ACAGGAGATAATGTTTCGGATGATG | E1：79-103 | 110 bp | 55 |
|  |  | R:TCCTGTTCCGCCTGACTGC | E1：188-170 |  |  |
| HPV-39 | CCCTAACTCTCAACCAGCACCCCTTTCAC | F:TCTAGATTATTAACAGTAGGACATCCA | L1：118-144 | 106 bp | 60 |
|  |  | R:CGCGAAATACCCTATATTGATATGCA | L1：223-198 |  |  |
| Cy5 | CTCTTAGTCACTTCCCCTTGGTTCTCTCTAC | Universal probe |  |  |  |
| HPV-59 | CTCTTAGTCTGTTCCCCTTGGGACTCTCTAC | F:ACTACTCGCAGCACCAATCT | L1：1006-1025 | 105 bp | 50 |
|  |  | R:TTCCTCCACATGTCTGGCAT | L1：1110-1091 |  |  |
| HPV-68 | CTCTTAGTCACTTCCCGTTGGTACTCTCTAC | F:GCCATGTTAGATGACGCAACA | E1：1525-1545 | 107 bp | 56 |
|  |  | R:AGGTGTCTGTGTTTTCTATCTAAACT | E1：1631-1606 |  |  |
| HPV-56 | CTCTTAGTCACTTGCCCTTGGTTCTCTCTAC | F:ACTGGGCACTAGGTCAAAGC | L1：1407-1426 | 105 bp | 62 |
|  |  | R:ACAACAACACACTACCGCCT | L1：1511-1429 |  |  |

Supplementary Table 3 2D PCR final reaction system

| Componentt | Volume |
| --- | --- |
| 10×Buffer（Mg2+ free） | 2.5 μL |
| 25 mM MgCl2 | 1.5 μL |
| 4×2.5 mM dNTPs | 0.7 μL |
| 5 U/μL HS Taq DNA polymerase | 0.5 μL |
| 10 μM FAM fluorescence probe | 0.4 μL |
| 10 μM VIC fluorescence probe | 0.3 μL |
| 10 μM CY5 fluorescence probe | 0.3 μL |
| HPV16-F15 | 0.2 μL |
| HPV18-F1 | 0.1 μL |
| HPV33-F10 | 0.1 μL |
| HPV58-F2 | 0.1 μL |
| HBB-F6 | 0.1 μL |
| HPV39-V8 | 0.1 μL |
| HPV45-V2 | 0.2 μL |
| HPV51-V7 | 0.2 μL |
| HPV52-V1 | 0.1 μL |
| HPV56-C9 | 0.2 μL |
| HPV59-C4 | 0.1 μL |
| HPV68-C7 | 0.1 μL |
| HPV16-R | 0.8 μL |
| HPV18-R | 0.8 μL |
| HPV33-R | 0.6 μL |
| HPV58-R | 0.6 μL |
